# Real-Time Detection and Quantification of Rail Surface Cracks Using Surface Acoustic Waves and Piezoelectric Patch Transducers

**DOI:** 10.3390/s25103014

**Published:** 2025-05-10

**Authors:** Mohsen Rezaei, Sven Eck, Sebastian Fichtenbauer, Jürgen Maierhofer, Reinhard Klambauer, Alexander Bergmann, David Künstner, Dino Velic, Hans-Peter Gänser

**Affiliations:** 1 Materials Center Leoben Forschung GmbH (MCL), 8700 Leoben, Austria; 2 Institute of Electrical Measurement and Sensor Systems, Graz University of Technology, 8010 Graz, Austria; 3 Voestalpine Rail Technology GmbH, 8700 Donawitz, Austria; 4 Voestalpine Signaling Austria GmbH, 8740 Zeltweg, Austria

**Keywords:** rail monitoring, surface acoustic waves, piezoelectric transducers, surface defects, rolling contact fatigue, crack quantification

## Abstract

This paper presents a novel wayside rail monitoring system for real-time detection and quantification of rail surface cracks with sub-millimeter precision. The core innovation lies in mounting piezoelectric transducers on the web of the rail—an unconventional and practical location that avoids interference with wheel passages while enabling continuous monitoring in real-world conditions. Moreover, to directly quantify crack depth, a customized signal processing pipeline is developed, employing surface acoustic waves (SAWs) and incorporating a parallel reference transducer pair mounted on an undamaged rail section for calibration. This auxiliary pair provides a real-time calibration baseline, improving measurement robustness and accuracy. The method is experimentally validated on rail samples and verified through metallographic analysis. This approach enables condition-based maintenance by improving detection accuracy and offers the potential to reduce operational costs and enhance railway safety.

## 1. Introduction

The U.S. Department of Transportation has identified surface defects, such as head checks (HCs), as a significant risk factor for rail failure. A major contributor to these surface defects is rolling contact fatigue (RCF), which occurs due to wheel–rail contact [1]. Fatigue-related issues account for 50–90% of mechanical failures in railways [2]. Rail monitoring is typically conducted using either track recording vehicles (TRVs) equipped with sensors or fixed sensor setups installed on vulnerable track sections. While both approaches have advantages, fixed setups enable continuous monitoring. The presented approach in this paper addresses the gap in cost-effective real-time systems for wayside rail monitoring by using piezoelectric transducers and ultrasonic waves to detect and quantify HCs.

Various methods and technologies exist for non-destructive testing (NDT) of rails, crack detection, and rail health monitoring, many of which have been comprehensively reviewed in [3,4]. A selection of the most commonly employed methods is briefly outlined, as follows.

One commonly used approach is visual inspection, which employs high-speed cameras mounted on train recording vehicles traveling along the track. These cameras capture detailed rail images, which are subsequently processed using image processing techniques to identify defects such as cracks, corrosion, rail gaps, missing bolts, and wear.

Eddy-current testing (ECT) employs two coils to induce and sense alternating magnetic fields near the surface of conductive materials. Surface defects disturb the magnetic field within the material, resulting in changes in the impedance of the magnetic circuit [3,5]. These impedance variations can be correlated with defect characteristics.

Similarly, magnetic flux leakage (MFL) is another method that, unlike ECT, uses DC magnetic fields [3,6]. It detects changes in the magnetic permeability of ferromagnetic surfaces caused by flux leakage due to defects, and the analysis of these changes provides insights into defect characteristics.

Another innovative technique for NDT of rails, proposed and implemented by the authors in [7,8,9], is a portable scanning inductive thermography setup. The proposed system consists of a movable table frame equipped with an induction coil and a thermography camera, which can be manually operated along the track. This method generates short heat pulses on top of the rail by inducing high-frequency eddy currents on the rail surface. The presence of cracks and defects causes a non-uniform heat conduction across the surface. Using the thermal camera, slight temperature disturbances can be detected and correlated to surface defects.

In [10], the authors employed a hybrid approach combining fixed strain gauges attached to the rail web and a portable laser profiling device to monitor rail defects, including plastic deformation, wear, and RCF, over a 30-month period. Railway turnout profiles were recorded at regular intervals of 3 to 6 months using laser scanning, enabling precise tracking of geometric changes and material degradation.

Another popular technology, which is also used in the present contribution, is ultrasonic waves. A wave is generated by a transducer and propagates across the surface (or inside the material). When traveling on the surface of a specimen, like at the frog of the turnout, the wave is disturbed upon encountering a crack. Measuring the reflection, transmission, and refraction of the wave can reveal information about the crack characteristics [3,11]. Ultrasonic testing has seen continuous development in recent years. A recent review on the application of ultrasonic technology for detecting rail defects can be found in [12]. A surface acoustic wave (SAW) is a type of ultrasonic wave that propagates along the surface of a material [11,13]. Various excitation sources can generate these waves, including piezoelectric transducers [14,15], electromagnetic acoustic transducers (EMATs) [16], and laser-based methods [3]. Additionally, spark discharge near the surface has been explored as an excitation mechanism (e.g., [17]).

The transducers used for generating SAWs can be either portable—facilitating periodic inspections of critical rail sections—or fixed installations for continuous structural health monitoring. In this research, we propose a novel (fixed) wayside condition monitoring (CM) concept for the rail.

Despite advances in NDT technologies, many existing approaches face significant limitations for long-term, reliable wayside rail monitoring. Laser-based or thermography methods often require bulky setups and are sensitive to environmental disturbances such as vibrations. Others, like eddy-current and MFL systems, may suffer from limited penetration depth or complex deployment. Moreover, scalability remains a major bottleneck due to high operational costs and maintenance complexity.

A promising solution to the challenges of cost, complexity, and scalability in wayside rail monitoring is the use of piezoelectric patch transducers. In this work, these transducers are employed in a fixed configuration to generate both surface acoustic waves (SAWs) and bulk waves—the former for detecting surface cracks and the latter for calibration. Piezoelectric transducers offer key advantages, including low cost (typically under USD 100), compact size, robustness, and the ability to operate over a broad frequency range, making them ideal for long-term monitoring. When coupled with remote acquisition and processing systems, they enable fully automated, real-time inspection without the need for frequent manual intervention.

The authors have previously demonstrated the feasibility of using piezoelectric transducers for rail crack detection [11], and later introduced a signal processing method to compute a damage index (DI) for crack monitoring [18]. In the current study, these earlier concepts are significantly extended by introducing three major innovations: (1) an improved web-mounted installation that is optimized for real-world use without interfering with passing wheels; (2) the introduction of a parallel reference sensor pair mounted on an undamaged rail segment to provide real-time calibration and increase measurement robustness; and (3) a refined signal processing pipeline that enables direct estimation of crack depth, going beyond DI-based health metrics to provide more actionable insights for predictive maintenance.

The following sections present these innovations in more detail, along with the technical background and the key challenges addressed in implementing this practical wayside rail monitoring system.

Implementing a fixed piezo-transducer setup for rail structural health monitoring presents several challenges, most of which are addressed in this research. One of the primary challenges is the transducer placement. Most existing studies on SAW with a pitch–catch (sender–receiver) configuration are implemented on simple, straight specimens, as seen in [19], or alongside the rail on the web or bottom [20,21]. In such setups, the shortest distance between the sender and receiver follows a direct path, ensuring minimal reflections and interference. This allows for a clear identification of the expected SAW arrival time. While these setups effectively detect defects along the rail between the sender and receiver, they are unable to capture defects on the rail head near the running gauge.

To monitor defects on the head and near the running gauge, the sensor pair should ideally be installed close to this region. However, placing transducers in these positions is impossible for field deployment, as they are directly in the wheel’s path. Instead, transducers must be mounted on both sides of the rail web, requiring SAW to propagate over the rail head, a complex geometry with curves and sharp edges. This configuration introduces significant wave scattering, leading to multiple reflections and interference, thereby complicating the detection of the primary wave. Additionally, the increased distance between transducers results in high signal attenuation, which generally increases with frequency [22]. To address this, the research optimizes the frequency range, ensuring a broad excitation spectrum while minimizing the overlap between scattered waves and the expected SAW at arrival time.

Another challenge is the measurement of the transmission coefficient. Since the transducers are far from each other and from the crack, direct measurement is infeasible. The transmission coefficient, by definition, requires the incident and transmitted amplitudes to be recorded immediately before and after the crack. To overcome this, a parallel sensor pair is installed to provide a baseline measurement over an undamaged section. The signal captured from this pair is used as a damage-free reference, allowing changes on the damaged side to be detected by comparison. Moreover, this pair can be used as a reference for calibrating the environmental effects. This setup also supports improved consistency in measurement, as both sensor pairs are similarly exposed to external influences, such as temperature, helping to stabilize the calibration process.

Signal analysis in such a system also poses challenges. In practical applications, the received signal is highly affected by noise and interference, making it difficult to isolate the relevant features. To address this, a customized signal processing pipeline is developed, specifically designed for the conditions of this setup, ensuring reliable feature extraction from the raw signals.

Beyond software solutions, a dedicated hardware infrastructure has been developed to support the system. A piezo driver unit in combination with a data acquisition system is designed and implemented to amplify both the transmitted and received signals. Additionally, a piezo switching unit (PSU) is developed to enable seamless switching between different transducer pairs. The PSU module improves system flexibility by allowing operation with four piezoelectric transducers instead of two, dynamically alternating between transducer pairs.

Experimental validation on a test rig, supported by micrographs, confirms that the proposed setup accurately traces crack depth evolution, demonstrating its applicability to real-world rail monitoring.

## 2. Materials and Methods

To realize this monitoring concept, a set of customized hardware and software needs to be designed, connected, and adjusted to work together. The following sections describe the various implemented parts and methods created to form a unit for generating and measuring SAW, an analysis of the recorded signals and extraction of the relevant signal features, and finally verification by means of full-scale tests on a wheel–rail test rig.

### 2.1. System Design

**Figure 1:**
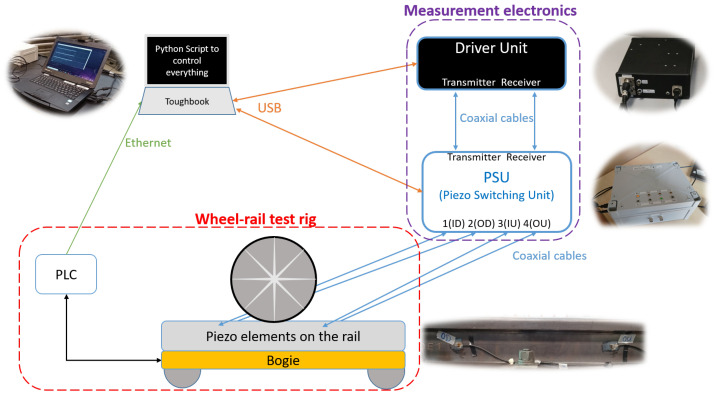
Overall testing system configuration.

The overall configuration of the test and measurement system and the connection between its components are shown in Figure 1. To evaluate the system performance, it was set up at the voestalpine wheel–rail test rig site in Donawitz, Austria. The test rig is described in detail in [23]. The monitoring system proposed in the present investigation integrates four piezoelectric transducers mounted on the rail web using adhesive. Two monitor the position on the rail where head checks are generated, while the other two are mounted outside the length the wheel runs over the rail and serve as reference signals. The transducers are connected through a PSU for flexible sender–receiver configurations; the signals are processed in real-time on an industrial laptop “ToughBook” laptop (Panasonic corporation, Kadoma, Japan). The PSU itself is connected to the driver unit, which serves as the amplifier of the sender/receiver piezos and is connected to the laptop. The laptop is also connected to the PLC unit of the test rig via a LAN.

As described in [18,23], the test rig uses a bogie (carriage), on which the rail sample is installed and fixed. A wheel actuated by a hydraulic cylinder can move up and down and touch the rail or detach from it with vertical force of up to 50 tons and lateral force of up to 10 tons.

The signals generated by the software are amplified via the driver unit and transmitted to/from the required transducers through the PSU. The post-processing signal pipeline then analyzes these data to estimate the crack depth. A detailed explanation of the system’s operation is provided in the following subsections.

#### 2.1.1. Transducers

Piezoelectric transducers utilize the electromechanical properties of piezoelectric materials to function as both actuators and sensors. A detailed description of these transducers is provided in [24].

For this project, an extensive investigation was conducted to identify suitable transducers and couplants (adhesives) for the intended application. Piezoelectric transducers were selected as an appropriate solution due to their compact size, broad frequency range, versatile design, cost-effectiveness for large-scale implementation, and ease of driving and installation. The transducer employed in this study is a barium titanate (BaTiO_3_) piezocomposite, model “P-876 DuraAct SP1” (PI Ceramic GmbH, Lederhose, Germany). Detailed specifications are available on the manufacturer’s website [25] and in a recent review on ultrasonic guided wave sensors for structural health monitoring applications [26]. Key characteristics of this transducer are also summarized in Table 1.

With regard to the adhesive, the long-term performance of various commercially available products was evaluated by comparing the surface acoustic wave (SAW) peak amplitude over several months. Based on these results, adhesive Z70 (Hottinger Brüel & Kjaer GmbH, Darmstadt, Germany) [27] was selected, as it demonstrated excellent durability as well as consistent signal transmission behavior over time. This adhesive has also been proven effective in strain gauge applications on railway tracks, where it has remained functional for several years.

In typical applications, a single pair of piezoelectric transducers is used as the sender and receiver. However, this configuration makes it difficult to distinguish signal changes caused by environmental effects (e.g., temperature fluctuations, wear) or aging of the adhesive and transducers from those resulting from actual changes in the specimen, such as crack development.

To address this issue, a second transducer pair is installed in parallel at a location further along the rail that is not prone to damage. This auxiliary pair serves as a calibration reference while the main pair is used for crack depth measurement. Since both pairs use the same transducer and adhesive type and are located close to each other, they are expected to experience similar environmental and aging effects. By compensating for the changes observed in the calibration pair, the main pair can more accurately reflect damage-related variations.

Although additional reference pairs could be installed to further improve calibration accuracy, this would increase system complexity, cost, and maintenance requirements. As a compromise between accuracy and practicality, four transducers were selected as the optimal configuration.

**Figure 2:**
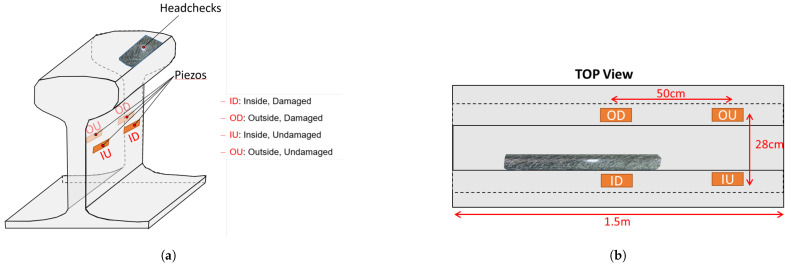
Piezo network configuration: (**a**) 3D view; (**b**) top view.

Subsequently, four transducers were configured as a network and installed on the specimen, as illustrated in Figure 2a. The transducers were mounted on the web of the rail. While this placement complicates signal processing compared to mounting them on the rail head, it is crucial for real-world applications, where a passing wheel would otherwise collide with transducers installed on the rail head.

As described above, in the proposed configuration one pair of transducers is positioned in a region prone to head checks and cracks, ensuring maximum exposure to potential damage (ID–OD pair shown in Figure 2b). The other pair is installed in a region outside the zone of wheel–rail contact, which experiences no damage and serves as a reference for signal calibration and assessing the extent of damage. By combining data from both sets of transducers, deviations from the normal condition can be quantified and correlated with the corresponding crack depth, providing a reliable measure of the rail’s structural health. Furthermore, the proposed configuration enables the measurement of sound velocity in the material under various environmental conditions. By knowing the fixed distance along the rail between specific transducers, such as OD and OU (as shown in Figure 2b), calculations of sound speed can be performed.

#### 2.1.2. Sender/Receiver Unit

**Figure 3:**
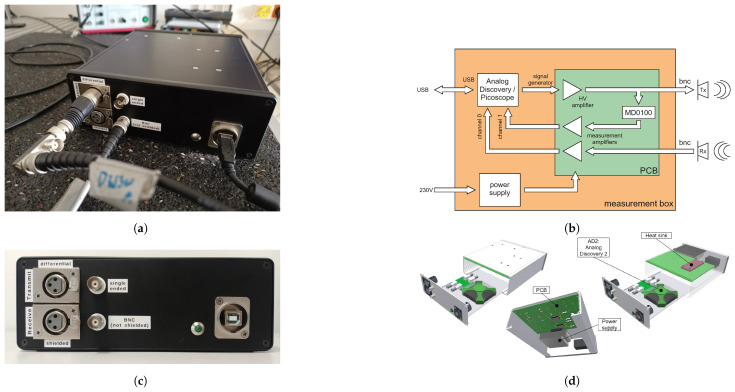
Driver unit: (**a**) overview; (**b**) schematic block diagram; (**c**) front panel; (**d**) exploded view.

The driver or sender/receiver unit serves as the central unit for transmitting and receiving signals to and from the piezoelectric transducers while also amplifying these signals. It functions as the driver for the piezo transducers (Figure 3a). At the core of this system is the Analog Discovery 2, a versatile digital oscilloscope and waveform generator from DIGILENT [28]. This device features two input channels with a 100 MS/s sampling rate, allowing it to capture signals up to 30 MHz and amplitudes of ±25 V with a maximum resolution of 14 bits. Additionally, it includes two 14-bit signal generator output channels capable of producing arbitrary signals up to a frequency of 12 MHz with maximum amplitudes of ±5 V.

Since the piezoelectric transducers require a minimum voltage of approximately 20 V to generate effective vibrations and a low output impedance to drive their capacitance in the MHz range, external signal amplification is necessary. Additionally, the signal level of the piezo receiver is typically weak and requires amplification. To address this, a custom-designed PCB with input and output operational amplifiers was developed to amplify both the transmitted and received signals. Furthermore, the internal circuit is designed so that the sender transducer can also measure the received signals, enabling the measurement of SAW reflections.

Heat sinks attached to the circuit and housing dissipate heat during operation and extended use, ensuring that all components function within their specified temperature ranges. An integrated power supply provides the required DC voltage to the custom PCB, which in turn generates the necessary voltage levels for the input and output amplifiers. The block diagram of the designed circuit is presented in Figure 3b.

As shown in Figure 3b, the embedded oscilloscope serves as the core of the system, handling both signal generation and acquisition. It connects to a PC via a USB to receive the user-defined excitation signal and transmit recorded data. The signal generator output of the oscilloscope is connected to the output amplifier circuit, which boosts the voltage to levels suitable for exciting the transducers. This amplified signal is then routed to the “Transmit” connectors on the front panel (see Figure 3c), where the actuator transducer is connected. Internally, this output path is also routed back to channel 1 of the oscilloscope through an overvoltage protection circuit and an additional amplifier, enabling the system to capture and amplify the reflected wave signal.

On the input side, the receiver transducer is connected to the “Receive” port on the front panel. The signal from this port is passed through a dedicated input amplifier stage and then fed to channel 0 of the oscilloscope for acquisition.

This configuration ensures reliable excitation and reception of signals while protecting the acquisition hardware from potential voltage spikes.

The integrated power supply unit delivers the required voltage levels to various components of the circuit, including ±5 V, 12 V, and ±20 V.

As discussed earlier, this unit has the flexibility of working with a wide range of signal settings. In this work, tone burst excitation signals (a sinusoidal signal with fixed amplitude and a limited number of periods) with a frequency range of 1 to 3 MHz are used. The duration of the burst is considered to be 5 full periods of the corresponding signal. A sample rate of 33 MS/s is used to ensure the signal features are captured properly.

The unit is enclosed in a durable housing with a front panel featuring various interfaces. It includes two standard BNC connectors for single-ended-mode sender/receiver signals, as well as two XLR connectors designed for differential mode. The differential mode drives the transmitting piezo with an amplitude of ±40 V. Additionally, the front panel features a USB interface for connecting the unit to a PC. These interfaces are illustrated in Figure 3c.

An exploded 3D view of the device is provided in Figure 3d, showing the main components of the system. These include the compact oscilloscope with its BNC connector adapter, the PCB for the peripheral circuitry (amplifiers, protective components, and passive elements), and the power supply circuit.

The driver unit is designed to connect to a single transducer pair (sender/receiver). However, certain applications require driving a network of piezoelectric transducers and switching between sender and receiver configurations. As stated earlier, an intermediary PSU was designed and implemented to address this issue.

**Figure 4:**
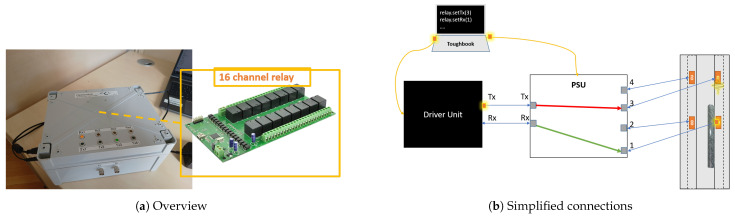
PSU: (**a**) overview; (**b**) simplified connections.

As illustrated in Figure 4, this unit incorporates a 16-relay module from Numato Company [29], shown in Figure 4a. The PSU features four BNC inputs, connected to the piezos mounted on the specimen, and two BNC outputs, which interface with the driver unit for transmitting and receiving signals. The user can designate specific piezos as transmitters or receivers. A simplified block diagram of the unit is presented in Figure 4b.

### 2.2. Signal Processing Pipeline

The proposed solution comprises not only a dedicated hardware setup but also a robust post-processing framework, essential to interpret the recorded signals and extract quantitative features.

The recorded signals undergo a signal processing phase that includes the following general steps:

- Noise reduction through cross-correlation.
- Identification of arrival time intervals.
- Estimation of damage index (DI) or crack depth by calculating transmission coefficients.

The plug-and-play design of the system and the program simplifies the role of the technician, requiring only the input of sensor distances for setup.

To achieve these objectives, two custom-designed signal processing pipelines are introduced. The first pipeline (Section 2.2.1) derives a DI by quantifying the deviation of the recorded signal from the ideal damage-free condition. The present paper provides a brief summary of the first method, with a stronger emphasis on the second pipeline (Section 2.2.2), which directly estimates crack depth.

#### 2.2.1. Deviation-Based Signal Processing Pipeline

**Figure 5:**
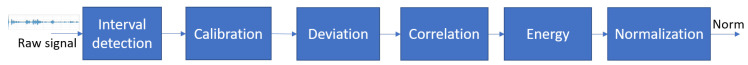
Deviation-based signal processing pipeline.

In a previous work by the authors [18], this method was disclosed in greater detail. It provides a quantitative index representing the deviation of the signals from a baseline (damage-free rail). This index enables rail operators to set thresholds for triggering preventive maintenance when exceeded. The pipeline consists of six high-level signal processing blocks, as illustrated in Figure 5.

**Figure 6:**
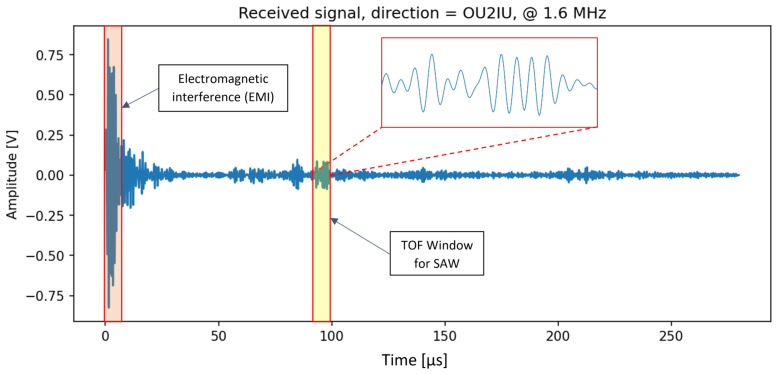
A typical received raw signal, with the approximate interval of the expected SAW arrival highlighted and magnified for closer inspection.

Each block in the pipeline performs a specific function, as detailed below:

- Interval detection:
  The received signal often exhibits a complex structure, containing noise, interference, and a combination of SAW and bulk waves. A typical received signal is shown in Figure 6. As seen, the signal has a weak amplitude and is influenced by various sources of noise and interference. To isolate the SAW component, a time-of-flight (ToF) analysis is conducted to estimate its expected arrival interval. In this case, a Rayleigh wave velocity of approximately 3000 m/s in steel is assumed, based on the material’s Young’s modulus and Poisson’s ratio. Given a transmitter–receiver spacing of 28 cm, the estimated time of flight is around 93 μs. Accordingly, a time window centered around this value—i.e., 93 μs ± (signal width/2)—is selected to accommodate uncertainties and ensure sufficient signal content for subsequent analysis, such as cross-correlation.
  This interval is visually marked and magnified in Figure 6 for clarity. It may be observed that a wave packet appears to arrive at nearly zero microseconds, which is not physically realistic. This early signal, also highlighted in the figure, is attributed to electromagnetic interference (EMI) between the sender and receiver ports, occurring at the moment the excitation signal is applied to the sender. The presence of such EMI is also indicated in the measurement results shown in [30] and further acknowledged in [31]. It is important to note that no additional excitation is applied to the sender port during the remaining signal duration, so no further EMI is expected. Although other wave modes, such as bulk waves, may be present in the time window following the EMI, the actual surface acoustic wave (SAW) packet arrives within the expected TOF window. Therefore, the EMI can be excluded, and the signal processing focuses only on the valid TOF interval.
- Calibration:
  Signal amplitudes can vary between cycles due to factors unrelated to crack development, such as adhesive aging or temperature fluctuations. To mitigate these effects, the algorithm calibrates the SAW signals using parts of the signal that remain unaffected by crack depth.
- Deviation calculation:
  Even within the expected SAW interval, the signal may contain bulk waves, electronic noise, and harmonics. These components often exhibit consistent patterns across cycles. By taking a snapshot of the signal at cycle zero, the algorithm calculates deviations for each subsequent cycle, effectively canceling out the unwanted components. The remaining deviation signal, in contrast, will primarily contain information related to crack depth evolution.
- Correlation:
  Despite previous preprocessing, the signal may still contain unwanted harmonics and noise at frequencies other than the excitation frequency. By correlating the processed signal with the transmitted signal, the algorithm acts as a matched filter, isolating the main frequency component for further analysis.
- Energy calculation:
  While many studies focus on peak amplitude, this approach considers the broader effect of the signal over a time interval. This is particularly important when using piezoelectric transducers, whose dimensions are comparable to the wavelength of the excitation, and excitation via burst signals extending over finite time. Instead of focusing on a single time point, the algorithm calculates the root mean square (RMS) value over the period of time the SAW takes to travel across the sensor, which correlates directly with crack depth.
- Normalization:
  The final output is an absolute value that becomes meaningful only when compared to a baseline measure. To achieve this, the calculated value is normalized with respect to the corresponding value at cycle zero (damage-free status).

The result is a normalized value, called the DI, that quantifies how much the signal has deviated from ideal, crack-free conditions. Based on this value, the operator can set an experimental threshold. Maintenance is triggered to address the detected damage if the DI exceeds this threshold.

#### 2.2.2. Transmission Coefficient Mapping Signal Processing Pipeline

Although the first algorithm provides a robust method for predictive maintenance scheduling, it is often more meaningful for rail operators to obtain a direct estimate of crack depth. To address this need, a second algorithm was developed and implemented.

This algorithm involves two primary steps: first, calculating the transmission coefficient for each measurement; and second, estimating the corresponding crack depth using a reference model or relation between the transmission coefficient and crack depth.

**Figure 7:**
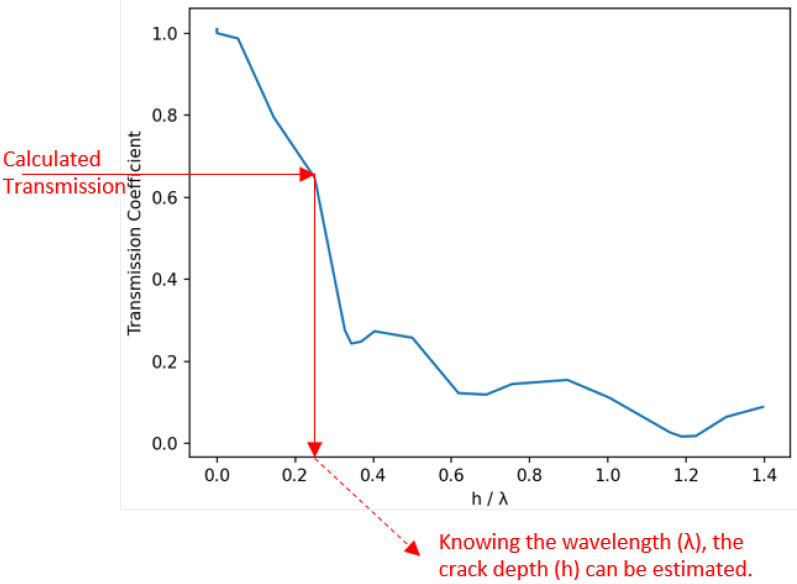
Transmission coefficient mapping method, using transmission coefficients of Rayleigh waves (the graph is reproduced from data shown in [32]). *h* is the crack depth and λ represents the signal wavelength.

For the second step, Achenbach [32] calculated various coefficients for Rayleigh waves, including transmission and reflection coefficients, as they propagate on the surface of a specimen and interact with a surface crack. Their results are presented as a diagram of transmission coefficient vs. crack depth normalized by wavelength, shown in Figure 7. From this diagram, the ratio of crack depth vs. wavelength can be determined if the transmission coefficient is known. Given that the excitation signal’s frequency and wavelength are known, the crack depth can then be calculated accordingly.

The first step involves determining the transmission coefficient of the signal, defined as

(1) $$T=\frac{A_{T}}{A_{I}}$$

where *T* is the transmission coefficient, $A_{T}$ is the amplitude of the transmitted signal (after the crack), and $A_{I}$ is the amplitude of the incident signal (before the crack). This requires measuring signal amplitudes immediately before and after the crack using equipment like a laser vibrometer. While feasible for laboratory experiments and portable or mobile setups such as TRVs, this method poses challenges for fixed installations.

For piezoelectric transducers, which are fixed on the rail web at a defined distance from each other (approximately 28 cm when measured along the contour of the rail profile), measuring $A_{I}$ and $A_{T}$ directly near the crack is impractical. Cracks typically occur on the rail’s running gauge, far from the transducer locations, as depicted in Figure 2a. Installing additional transducers before and after the cracks is also infeasible. Instead, an alternative setup is proposed, consisting of two pairs of transducers installed at the undamaged and damaged sections of the rail, as shown above in Figure 2a. In this configuration, the undamaged section is used to calculate the incident amplitude $A_{I}$, while the damaged section provides the transmitted amplitude $A_{T}$. Theoretically, the only difference between the signals from the undamaged and damaged sections arises from the development of head checks. Thus, the ratio of these signals reflects the transmission coefficient, which quantifies how much of the signal is transmitted relative to the crack depth.

**Figure 8:**
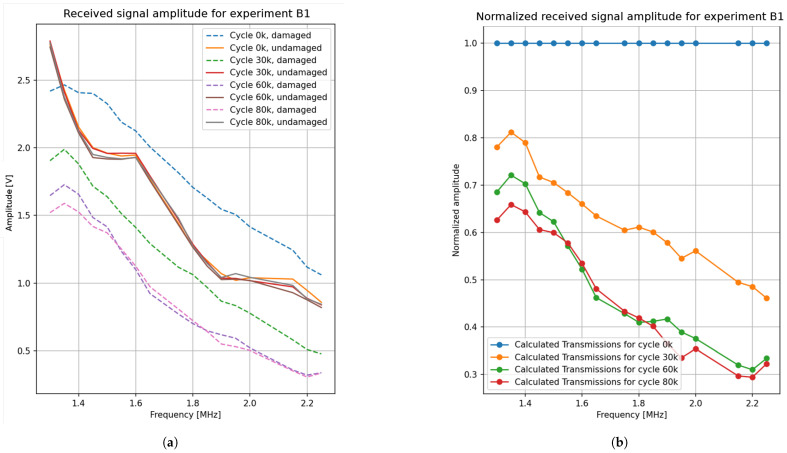
Received signal amplitudes on the damaged and undamaged sections at various frequencies over the cycles: (**a**) Received amplitudes in volts [V]; (**b**) received amplitudes normalized with respect to the undamaged section.

This concept is illustrated in Figure 8, which presents the amplitudes of the received signals for the damaged and undamaged sections at various excitation frequencies for a sample experiment. Figure 8a displays the raw amplitudes for both sections. As shown, the amplitudes in the undamaged section remain highly consistent over the measurement cycles (0, 30, 60, and 80 k), with minimal variation. In contrast, the damaged section exhibits significant changes over cycles, influenced by the development of the crack depth. In addition, the amplitudes on the damaged section generally decrease as the number of cycles increases, indicating that the crack is growing and obstructing a larger portion of the SAW. These amplitudes are then normalized with respect to the corresponding amplitudes at the undamaged section and the damaged section at cycle zero (no damage), as shown in the graph in Figure 8b. As observed, the normalized values are smaller than 1, reflecting the effect of normalization. These normalized values correspond to the transmission coefficients and can be utilized for determining the crack depth, as previously discussed.

This idea effectively provides an affordable and robust implementation for a fixed monitoring system, offering a practical alternative to traditional methods.

**Figure 9:**
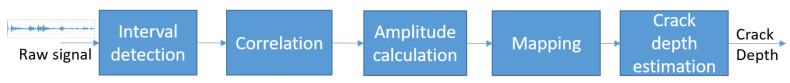
Transmission coefficient mapping signal processing pipeline.

The corresponding signal processing pipeline is illustrated in Figure 9. The implementation steps are described below:

- Interval detection:
  Identifies the relevant portion of the signal for analysis, as described in the previous section.
- Correlation:
  Cleans the signal by isolating the frequency component of the excitation signal, as discussed in the previous section.
- Amplitude calculation:
  The maximum amplitudes of the correlations from the previous step are determined, and their square root values are computed. The resulting values from the damaged and undamaged signals correspond to $A_{T}$ and $A_{I}$, respectively. The transmission coefficient is then calculated as the ratio of these two values. This process is repeated for all measurements taken at different excitation frequencies.
- Transmission coefficient mapping:
  The transmission coefficients, calculated using formula (1), at various frequencies are analyzed, and outliers are removed. Outliers are defined as transmission coefficients greater than 1 (unrealistic) or lower than 0.35. The latter threshold is based on the transmission graph in Figure 7, which shows that for transmission coefficients below 0.35 there is no monotonic relationship between the coefficient and the crack depth. After filtering, the corresponding crack depth is estimated for each valid transmission coefficient.
- Crack depth estimation:
  Since a single crack depth is estimated for each excitation frequency in the previous step, a distribution of crack depths is obtained. To remove any remaining outliers, a filtering process using the median absolute deviation (MAD) technique is applied. MAD is a robust statistical measure that calculates the median of the absolute deviations from the dataset’s median:
  (2) $$\mathrm{MAD}=\mathrm{median}(|{\mathrm{depth}}_{i}-\mathrm{median}\left({\mathrm{depth}}_{i}\right)|)$$

Unlike methods based on the standard deviation, the MAD is less sensitive to extreme values and provides a more reliable detection of outliers, especially in datasets with non-normal distributions or heavy-tailed behavior [33].

Outliers are identified as values outside the range median±c·MAD, where *c* is a constant scaling factor. In this project, c=3 is used. After removing these outliers, the filtered data are used to compute the final crack depth estimate.

### 2.3. Validation Setup

Testing was conducted at voestalpine Donawitz using a test rig designed to simulate wheel–rail interactions. The system was evaluated using pearlitic rail grades and a test rig setup targeted at the creation of gauge corner cracks, also known as HCs [23]. The results were validated through metallography and eddy-current testing. The overall configuration is depicted in Figure 1. As briefly described in Section 2.1, the specimen is mounted on the bogie, which moves forward and backward. During the forward motion, the wheel contact induces head checks over cycles, replicating realistic train wheel–rail interactions.

The excitation signals consist of bursts and wavelet-shaped pulses, each with 5 pulse widths. The frequency range generally spans from 0.5 MHz to 3 MHz, with step sizes of 100 kHz, or 50 kHz for higher resolution. Using the PSU, measurements are conducted across 8 distinct paths, including {OD ↔ ID, OU ↔ IU, OD ↔ OU, ID ↔ IU}, as explained and illustrated in Figure 2a. Among these paths, OD ↔ ID represents the primary signal paths, capturing the effect of the crack depth on the signal, while OU ↔ IU serves as the baseline, representing the crack-free zone. The remaining paths are used to verify the signal consistency.

Combining the frequencies and directions, approximately 256 distinct measurements are performed every 10,000-cycles. To reduce the effect of noise, each measurement is repeated 32 times, and the average value is calculated. In total, three separate experiments are conducted as part of this study. The total number of running cycles varies depending on the specific objective of each experiment. The following section presents the results from experiments with total cycle counts of 310,000, 80,000, and 30,000.

## 3. Results

### 3.1. Experimental Data

Various experiments were conducted in the test rig, testing different samples and configurations, to evaluate both provided signal processing pipelines.

#### 3.1.1. DI Calculation

**Figure 10:**
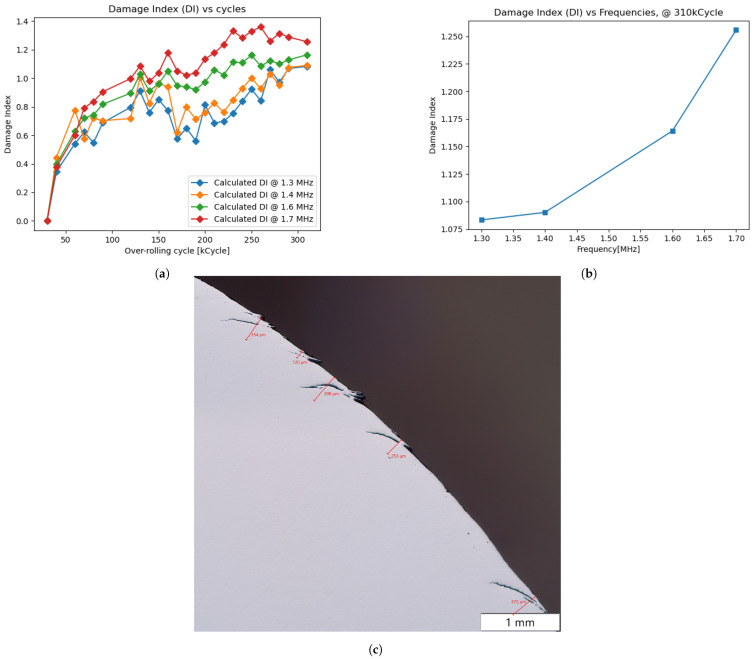
Results of experiment A: (**a**) for various frequencies; (**b**) for 310 k cycles; (**c**) metallography result after 310,000 over-rolling cycles.

To assess the DI, a test, referred to as experiment A, was conducted with 310,000 overcycles, taking measurements every 10,000 cycles. The DI, as defined in Section 2.2.1, was calculated for four frequencies (1.3, 1.4, 1.6, and 1.7 MHz), with the results presented in Figure 10a,b. In general, the DI increases progressively with the number of cycles for all frequencies, indicating crack growth, which obstructs signal transmission.

Higher frequencies exhibited greater DI values, reaching approximately 1 at the final stage, aligning with the transmission coefficient trends in Figure 7. The nearly linear DI progression, as seen, e.g., at 310,000 cycles in Figure 10b, correlates well with the theoretical near-linear behavior for $\frac{h}{\lambda }<0.3$ in Figure 7.

Metallography analysis after 310,000 cycles (Figure 10c) revealed a maximum crack depth of 398 µm and a mean depth of 295 µm. The progression from 0 µm at the start to a mean depth of 295 µm at the end demonstrates an agreement between the DI evolution and actual crack growth.

#### 3.1.2. Crack Depth Estimation

To verify the second signal processing pipeline, introduced in Section 2.2.2, additional experiments were conducted, and the crack depth at various cycles was measured using metallography for comparison. To assess the actual crack depth at multiple cycles, rather than just at the end of the experiment, the PLC program controlling the bogie and wheel movement was modified. Two experimental cases are presented here.

**Figure 11:**
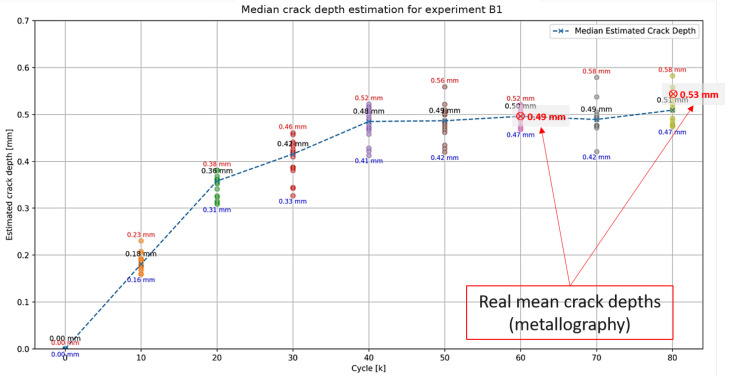
Estimated and actual crack depths for experiment B1.

The first of these experiments (referred to as experiment B1) was conducted up to 80 k cycles, with metallography performed on sample pieces at 60 k cycles and 80 k cycles to determine the true crack depth. The estimated crack depth predicted by the proposed method, along with the metallography results, is plotted in Figure 11. Each dot in the plot represents the estimated depth obtained at a certain frequency, as described in Section 2.2.2, resulting in a distribution of estimated crack depths for each cycle. The minimum, maximum, and median values of these distributions are indicated on the plot for each measurement set. As observed, the proposed method closely approximates the median crack depth.

**Figure 12:**
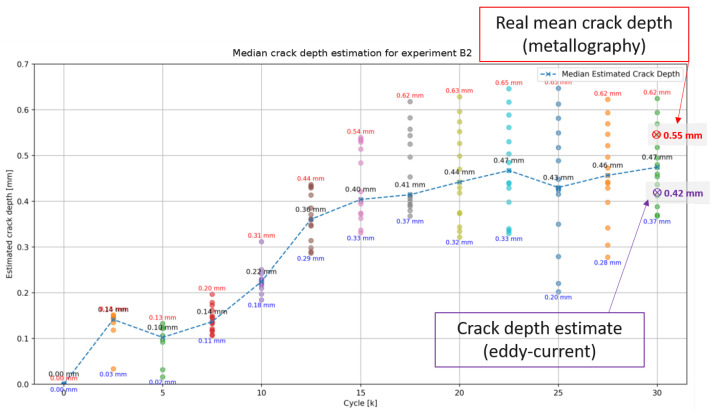
Estimated and actual crack depths for experiment B2.

In a subsequent experiment (experiment B2), a sample was tested up to 30 k cycles, with measurements taken every 2500 cycles for higher resolution. At the end of the experiment, the actual crack depth was measured via metallography. Additionally, an eddy-current test was performed to compare its crack depth estimate with those obtained using the proposed method. The results are illustrated in Figure 12.

During visual inspections every 2500 cycles, HCs gradually became visible after approximately 10,000 cycles, which qualitatively aligns with the crack estimation output. Regarding the final results, the estimated crack depth using the proposed method (0.47 mm) closely matches the real crack depth measured by metallography (0.55 mm). Notably, the proposed method provides a more accurate crack depth estimation than the eddy-current test (0.42 mm).

### 3.2. Performance Metrics

To evaluate the accuracy of the results, the estimated crack depths are compared with actual measurements obtained through metallography, as summarized in Table 2. The first three rows present the estimation results of the proposed algorithm, while the last row shows the results from the eddy-current test. As observed, the proposed algorithm closely matches the actual crack depths, with a prediction error not exceeding 15%, while the eddy-current test exhibits an error of 24%.

The average prediction error of the proposed method is calculated as the mean of the absolute percentage errors across all available the metallography comparisons shown in Table 2 (i.e., |+2%|, |−4%|, and |−15%|), resulting in an average absolute error of approximately 7%.

Moreover, the standard deviations associated with the estimated values encompass the measured depths, indicating both accuracy and consistency in the estimations.

## 4. Conclusions

This paper presents a novel, reliable, and cost-effective system for permanent real-time detection and quantification of rail cracks using SAW. The system consists of piezo transducers mounted on the rail web, a custom driver (sender/receiver) unit along with a PSU, and a laptop. All components are controlled and synchronized by a custom-developed Python V3.10 code running on the laptop. The dedicated signal processing pipeline enables the estimation of a DI and direct crack depth quantification from the received signals. Additionally, a reference sensor pair installed on an undamaged section allows for in situ calibration and improves robustness against environmental effects.

Experimental testing has demonstrated the system’s potential to improve rail maintenance practices by providing accurate and timely information on rail crack development. Test samples from various experiments were subjected to metallography and eddy-current testing to determine the actual crack depths. A comparison of the proposed method’s estimates with metallography reveals an average estimation error of approximately 7%. In real-world applications, due to practical factors such as measurement noise and environmental variations, an average error of around 15% can be expected. Despite these challenges, the system remains a reliable and practical solution for rail maintenance.

While with the present setup the proof of concept has been demonstrated on system level at laboratory scale, further steps are necessary to move forward towards a field demonstration. This includes measures for energy efficiency and energy harvesting, and extension to monitoring of multiple cross-sections along an extended length of the rail.

## Figures and Tables

**Table 1:** Specifications for P-876.SP1 patch transducer [25].

Specification	P-876.SP1
Dimensions L × W × T (mm)	16 × 13 × 0.5
Minimum lateral contraction (μm/m)	650
Relative lateral contraction (μm/m/V)	1.3
Operating voltage (V)	−100 to 400
Actuator type	Transducer
Piezo material	PIC255
Piezoceramic height (μm)	200
Electrical capacitance (nF)	8 (±20%)
Blocking force (N)	280
Operating temperature range (°C)	−20 to 150
Connector	Solderable contacts

**Table 2:** Comparison of measured and estimated mean crack depths (± STD) along with error percentages.

Test Run	Cycle [k]	Estimation Method	Measured Depth [mm]	Estimated Depth [mm]	Error [%]
B1	60	Proposed algorithm	0.49	0.50 _± 0.019_	+2
B1	80	Proposed algorithm	0.53	0.51 _± 0.032_	−4
B2	30	Proposed algorithm	0.55	0.47 _± 0.077_	−15
B2	30	Eddy current	0.55	0.42	−24

## Data Availability

Data are contained within the article.

## Abbreviations

The following abbreviations are used in this manuscript:

CM	Condition monitoring
DI	Damage index
ECT	Eddy-current testing
EMAT	Electro magnetic acoustic transducers
HC	Head checks
MAD	Median absolute deviation
MFL	Magnetic flux leakag
NDT	Non-destructive testing
PSU	Piezo switching unit
RCF	Rolling contact fatigue
RMS	Root mean square
SAW	Surface acoustic wave
ToF	Time of flight
TRV	Track recording vehicles
Glossary	
**CM**	condition monitoring. 2
**DI**	damage index. 8–10, 13, 14, 16
**ECT**	eddy-current testing. 2
**EMAT**	electromagnetic acoustic transducer. 2
**HC**	head check. 1, 13, 15
**MAD**	median absolute deviation. 13
**MFL**	magnetic flux leakage. 2
**NDT**	non-destructive testing. 1, 2
**PSU**	piezo switching unit. 4, 8, 13, 16
**RCF**	rolling contact fatigue. 1, 2
**RMS**	root mean square. 10
**SAW**	surface acoustic wave. 1–4, 7, 9–11, 16
**ToF**	time of flight. 9
**TRV**	track recording vehicle. 1, 11
